# SNHG12 downregulation induces follicular dysplasia by modulating the glycolysis of granulosa cell in polycystic ovary syndrome

**DOI:** 10.3389/fcell.2025.1585987

**Published:** 2025-05-23

**Authors:** Sisi Yan, Bing Qu, Yu Chen, Qiuji Wu, Jinli Ding, Hui Qiu

**Affiliations:** ^1^ Hubei Key Laboratory of Tumor Biological Behaviors, Department of Radiation and Medical Oncology, Hubei Cancer Clinical Study Center, Zhongnan Hospital of Wuhan University, Wuhan, China; ^2^ Department of General Surgery, Renmin Hospital of Wuhan University, Wuhan, Hubei, China; ^3^ Reproductive Medicine Center, Wuhan Children’s Hopital (Wuhan Maternal and Child Healthcare Hospital), Tongji Medical College, Huazhong University of Science and Technology, Wuhan, China; ^4^ Reproductive Medical Center, Renmin Hospital of Wuhan University and Hubei Clinic Research Center for Assisted Reproductive Technology and Embryonic Development, Wuhan, China

**Keywords:** SNHG12, PCOS, granulosa cells, glycolysis, follicular dysplasia, HMGB1

## Abstract

**Introduction:**

Polycystic ovary syndrome (PCOS) is characterized by follicular dysplasia, with granulosa cells (GCs) glycolysis playing a pivotal role in this pathology. Although the involvement of long noncoding RNAs (lncRNAs) in diverse biological processes of PCOS has been well documented, the molecular mechanism of lncRNA small nucleolar RNA host gene 12 (SNHG12) in PCOS remains unclear.

**Methods:**

In this study, we measured SNHG12 expression in GCs of PCOS patients and healthy controls using RT-PCR and performed correlation analysis between SNHG12 expression and glycolytic markers. Using granulosa-like tumor (KGN) cells, we investigated glycolytic capacity and examined the relationship among SNHG12, PTEN and HMGB1 through RNA immunoprecipitation (RIP) and chromatin immunoprecipitation (ChIP) assays. Finally, DHEA-induced PCOS mice was constructed using SNHG12 adenovirus to explore its role in PCOS.

**Results:**

SNHG12 expression was significantly downregulated in GCs from PCOS patients compared with healthy controls, and showed positive correlation with glycolytic markers. Functional studies demonstrated that SNHG12 knockdown impaired glycolysis in KGN cells, while SNHG12 overexpression partially restored glycolysis. Furthermore, SNHG12-induced glycolysis affected apoptosis of KGN cells, which mediated follicular dysplasia through lactate production and apoptotic pathways. *In vivo*, adenovirus-mediated SNHG12 overexpression alleviated the symptoms of PCOS mice. Mechanistically, RIP and ChIP assays revealed that SNHG12 interacts with HMGB1 and inhibits PTEN transcription by preventing HMGB1 from binding to the PTEN promoter, thereby promoting glycolysis in KGN cells.

**Conclusion:**

Our findings collectively demonstrate that the SNHG12/HMGB1/PTEN axis serves as a novel regulatory mechanism in PCOS by modulating glycolytic-mediated follicular dysplasia in GCs, offering a potential therapeutic target for PCOS.

## 1 Introduction

Polycystic ovary syndrome (PCOS) is one of the most prevalent endocrine and metabolic disorders among premenopausal women and is associated with clinical manifestations including acne, anovulatory infertility, and miscarriage (Goodarzi et al., 2011). PCOS is mainly characterized by ovulation disorders, polycystic ovaries, and hyperandrogenism, and is frequently accompanied by insulin resistance, metabolic syndrome, and an increased risk of cardiovascular complications (Escobar-Morreale, 2018). Although the complex of clinical manifestations and the heterogeneous of etiology in patients with PCOS, follicular dysplasia remains a typical feature of PCOS (Kulkarni et al., 2023). Nevertheless, the precise pathogenic mechanisms underlying PCOS development still remain elusive.

Emerging evidence highlights the crucial role of glycolysis in granulosa cells (GCs) in the pathogenesis of PCOS (Liu et al., 2024; Mu et al., 2023). It is widely recognized that oocytes mainly rely on GCs for glucose uptake due to their limited glycolytic capacity (Jiang et al., 2020). The impaired glycolysis in GCs leads to insufficient energy supply for follicular development and promotes the apoptosis of GCs, which has been identified as a key driver of follicular atresia in PCOS (Cui et al., 2024). Extensive clinical and experimental evidence has demonstrated that impaired glucose metabolism represents a hallmark feature of PCOS, with studies specifically identifying deficient glycolytic function as a key metabolic disturbance (Wang and Wu, 2020; Zhang et al., 2022). Consequently, dysregulated glycolysis in GCs significantly impacts both granulosa cells function and oocyte development, potentially contributing to follicular dysplasia in PCOS.

Long non-coding RNAs (lncRNAs), defined as non-coding transcripts longer than 200 nucleotides, have attracted considerable attention over the past decade due to their regulatory roles in various biological processes (Jandura and Krause, 2017). These molecules function as pivotal regulators in diverse pathophysiological processes, including epigenetic modulation, protein interactions, and transcriptional regulation (Herman et al., 2022; Quinn and Chang, 2016). Accumulating evidence indicates that dysregulation of lncRNAs contributes to metabolic dysfunction, endocrine disruption, altered proliferation and apoptosis of GCs in women with PCOS (Huang et al., 2021). For instance, lncRNA MALAT1 is highly expressed in the GCs of PCOS patients, and its knockdown has been shown to promote GC apoptosis, potentially affecting pregnancy outcomes in PCOS (Tu et al., 2022). Recently, glycolytic activity has emerged as a focal point in PCOS research, with studies such as that by Zhao et al. identifying lncRNA networks involved in PCOS-associated metabolic pathways (Zhao et al., 2021). Among these, the small nucleolar RNA host genes (SNHGs), an emerging class of gene transcription regulators (Xiao et al., 2023), including SNHG5 and SNHG7, have been reported to exert critical roles in PCOS (Aldakheel et al., 2021; Yang et al., 2024). Notably, SNHG12 has been identified as a glycolysis-related lncRNA in various diseases (Huang et al., 2024; Xia et al., 2021), and recent studies have suggested that it modulates the proliferation and apoptosis of granulosa cells by sponging miR-129 and miR-125b (Xuan et al., 2024). However, the specific role of SNHG12 in glycolysis during PCOS and its underlying mechanisms remain poorly understood.

In this study, we show that SNHG12 expression is markedly downregulated in GCs of PCOS patients and positively correlates with glycolysis-related gene expression. Functional studies identify SNHG12 as a key metabolic regulator in GCs that mediates follicular dysplasia in PCOS. Mechanistically, SNHG12 modulates the interaction between the transcription factor HMGB1 and the PTEN promoter, thereby suppressing the transcription of PTEN and participating in the pathogenesis of PCOS.

## 2 Materials and methods

### 2.1 Patients and tissue samples

Our study was conducted with the approval of the Institutional Review and Ethics Boards of Renmin Hospital of Wuhan University (Ethical Approval Number: WDRY2019-K077), and written informed consent was obtained from each participant. Primary ovarian granulosa cells (GCs) were collected from patients suffered with PCOS (*n* = 20) and healthy controls (*n* = 20). PCOS diagnoses were based on the 2003 Rotterdam Criteria (Rotterdam, 2004), and the baseline characteristics of the participants were listed in Table 1.

**TABLE 1:** Baseline characteristics of the study population (*n* = 20/group).

Characteristics	Control group	PCOS group
Maternal age (y)	30.7 ± 3.2	29.4 ± 2.6
Body mass index (kg/m^2^)	21.0 ± 2.3	23.9 ± 3.1*
Serum AMH (ng/mL)	4.1 ± 2.2	8.8 ± 1.4**
FSH (mIU/mL)	7.29 ± 2.33	5.83 ± 2.05
E2 (pmol/L)	188.21 ± 12.36	112 ± 10.64*
LH (mIU/mL)	3.05 ± 2.12	5.94 ± 2.68*

BMI, body mass index; AMH, Anti-mullerian hormone; FSH, follicle stimulating hormone; LH, luteinizing hormone; E2, estradiol. Data are presented as mean ± SD. *P < 0.05; **P < 0.01.

GCs were isolated from pooled follicular aspirates obtained during oocyte retrieval following a long stimulation protocol. Briefly, follicular fluid was centrifuged at 2,000 rpm for 10 min, and discarded the supernatant. The cell suspension was slowly added to 50% Percoll (Biosharp, Wuhan) and centrifuged at 1,800 rpm for 20 min. The intermediate layer containing granulosa cells was collected, and incubated with red blood cell lysis buffer (Biosharp, Wuhan) for 5 min, then washed with PBS and stored at −80°C for subsequent analysis.

### 2.2 Animals and experimental protocol

All *in vivo* experiments were approved by the Animal Care and Use Committee of the Wuhan University (Ethical Approval Number 20230909A). Twenty-one-day-old female C57BL/6J mice were obtained from the Animal Experiment Center of Wuhan University. The control group was injected with olive oil only (control group, *n* = 6). The PCOS mouse model was constructed as previously described (Yan et al., 2021), mice were injected subcutaneously with dehydroepiandrosterone (DHEA) (6  mg/100 g/d in olive oil) for 21 consecutive days (PCOS group, *n* = 6). For the SNHG12 study, DHEA-treated mice were injected with SNHG12-overexpressed adenovirus (Genechem, Shanghai, 10 μL 1E+10 PFU/mL) via the tail vein on the first day after DHEA treatment (OE-SNHG12 group, *n* = 6). During the treatment period, vaginal smears were collected daily beginning on the 10th day of the first DHEA injection until the end of the experiment. Three weeks after DHEA treatment, mice were euthanized under isoflurane anesthesia. One ovary from each mouse was dissected for PCR or Western blot, while the other ovary was fixed in formaldehyde solution for further examination.

### 2.3 Cell culture and treatment

The KGN cells were acquired from the Institute of Biochemistry and Cell Biology, and grown in DMEM/F-12 medium (Gibco, China) with 10% FBS (Gibco) at 37°C in 5% CO_2_. SNHG12-specific small interfering RNA (si-SNHG12) and a negative control shRNA (NC) were purchased from RiboBio (Guangzhou, China). Lentiviral vectors containing the pCDH-CMV-Human construct for SNHG12 overexpression was acquired from Genechem (Shanghai, China). Additionally, Vigene (Shandong, China) provided the empty vector control (NC), PTEN-overexpression plasmid (OE-PTEN), and HMGB1-overexpression plasmid (OE-HMGB1). Transfection of KGN cells with the aforementioned oligonucleotides and plasmids was performed using Lipofectamine 2000 reagent (Invitrogen).

### 2.4 Quantitative reverse transcription PCR (RT-PCR)

Total RNA was extracted from tissues and cells using TRIzol reagent (Invitrogen) following the manufacturer’s protocol. RNA was reverse-transcribed into cDNA using an mRNA Reverse-Transcription Kit (Takara, Japan), and cDNA was quantified by SYBR Green PCR Mix (Takara, Japan) using an Applied Biosystems 7,500 instrument. All samples were analyzed in triplicate, and relative gene expression levels were calculated using the 2^−ΔΔ^ Ct method. Actin was used as an internal control, and the primers were listed in Table 2.

**TABLE 2:** Oligonucleotide sequences and primer sequences for this study.

Gene	Primer sequence (5′–3′)
H-Actin	F: AACCGCGAGAAGATGACCCAG R: GTCACCGGAGTCCATCACGAT
H-HK2	F: CATCCAGAGGAGAGGGGACT R: TCATCGCCTTCCACCATGTC
H-LDHA	F: ATGGCAACTCTAAAGGATCAGC R: CCAACCCCAACAACTGTAATCT
H-PKM2	F: CTTGCAATTATTTGAGGAACTCCGC R: CACGGTACAGGTGGGCCTGAC
H-PTEN	F: CGTTACCTGTGTGTGGTGATA R: CTCTGGTCCTGGTATGAAGAATG
H-HMGB1	F: TATGGCAAAAGCGGACAAGG R: CTTCGCAACATCACCAATGGA
ChIP-1	
Site 1	F: TCAGTCCTTTGGCTTGCTCTTA R: ACTGGTTACACAAGCACCCACA
Site 2	F: GAGAACCGAGCTTGACTCCG R: AGGATCCCTGTGAGTGGGAC
Site 3	F: CCTCCCCTCGGTCTTCCG R: AAAGAGTCCCGCCACATCAC
Site 4	F: ATGCTCAGTAGAGCCTGCG R: CTCGGAAGACCGAGGGGA

### 2.5 Western blot

Total proteins were extracted from cells and tissues using established methods (Yan et al., 2023). Protein concentrations were determined using a BCA assay, and equal amounts of lysates were separated by SDS-PAGE before being transferred to PVDF membranes. The membranes were incubated overnight at 4°C with antibodies against Actin (Proteintech, Cat# 20536-1-AP, 1:3000), HK2 (Cat# 22029-1-AP, 1:2000), LDHA (Cat# 19987-1-AP, 1:2000), PKM2 (Cat# 15822-1-AP, 1:1000), Bcl2 (Cat# 68103-1-Ig, 1:2000), Bax (Cat# 50599-2-Ig, 1:2000), HMGB1 (Cat# 10829-1-AP, 1:1000) and PTEN (Cat# 22034-1-AP, 1:1000). After washing, the membranes were incubated with the secondary antibody for 1 h. The chemiluminescence (ECL; Bio-Rad, Hercules, CA, United States) was applied to observe. Actin was used as the loading control throughout the study.

### 2.6 Histochemistry (HE) and immunohistochemistry (IHC)

HE and IHC were performed according to previously described protocols (Yan et al., 2021). For IHC analysis, tissue sections were incubated with primary antibodies against PTEN (1:200) and HK2 (1:150), followed by appropriate secondary antibody detection. After counterstaining with hematoxylin, representative tissue sections were viewed, analyzed, and imaged under a light microscope (Nikon, Tokyo, Japan).

### 2.7 EdU and terminal deoxynucleotidyl transferase-mediated dUTP nick-end labeling (TUNEL) assay

The proliferation of KGN cells was assessed using an EdU cell proliferation kit (C0078S, Beyotime, China). After treatment, cells were incubated with serum-free DMEM/F12 medium containing 1:1000 EdU 2 h. For the TUNEL assay, ovarian paraffin sections were washed with PBS, fixed in 4% paraformaldehyde for 30 min and permeabilized with 0.1% Triton X-100 for 5 min. The sections were then incubated with the TUNEL detection kit (Beyotime, Shanghai) in the dark at 37°C for 30 min. Three randomly selected fields in each sample were analyzed and imaged.

### 2.8 RNA pulldown assay

The biotinylated oligonucleotide probe of SNHG12 was synthesized by GenePharma (Shanghai, China), with the antisense sequence of biotinylated SNHG12 serving as a control. As described in our prior study (Wang et al., 2024), SNHG12 probes were incubated with streptomycin magnetic beads (Thermo Scientific) for 1 h at room temperature. The resulting complexes were then incubated with cell lysates overnight at 4°C with gentle rotation. After washing five times with ice-cold wash buffers, the bound protein was eluted and analyzed by Western blot analysis. The sequences of the RNA pulldown probes were provided in Table 2.

### 2.9 RNA immunoprecipitation (RIP)

RIP assays were conducted to investigate the interaction between SNHG12 and HMGB1 protein using an RIP Kit (Millipore, Germany) according to the manufacturer’s protocol. Briefly, cells were lysed using complete RIP lysis buffer to preserve RNA-protein interactions. For immunoprecipitation, magnetic beads were incubated with HMGB1 or IgG antibody for 30 min. The antibody-conjugated beads were then added to the cell lysates and incubated overnight at 4°C with gentle rotation. The resulting complexes were extracted using Trizol reagent (Invitrogen, United States) and purified for RT-PCR assay to detect SNHG12 enrichment. The relative enrichment was calculated by normalizing to the IgG control group.

### 2.10 Luciferase reporter assay

Plasmids were constructed encoding the following reporter genes: the internal reference plasmid pRL-TK, P1 site of PTEN promoter, full-length wild-type (WT) PTEN promoter, mutated binding site one of PTEN promoter (GCGGGGGC→CCTTAA). For construction of the PTEN luciferase promoter, different fragments of human PTEN promoter DNA were PCR amplified and inserted into the pGL3-basic vector digested with the restriction enzymes KpnI and NheI. All constructs were verified by DNA sequencing. The above plasmids were co-transfected with either the SNHG12 overexpression plasmid, HMGB1 overexpression plasmid, or empty vector control using Lipofectamine 2000. After 48 h of transfection, cells were lysed and promoter activities were quantitatively measured using a Dual Luciferase Reporter Gene Assay Kit (Yeasen, China) according to the manufacturer’s protocol. Luciferase activity was normalized to the corresponding Renilla luciferase activity for each sample to account for transfection efficiency.

### 2.11 Chromatin immunoprecipitation (ChIP)

ChIP assay was performed using a ChIP Kit (ABclonal Technology, China) according to the manufacturer’s instruction. In brief, cells (1 × 10^7^) were fixed with 1% formaldehyde to crosslink for 10 min, followed by quenching with 125 mM glycine. Chromatin extracts containing DNA fragments were immunoprecipitated overnight using 2 μg of anti-HMGB1 antibody or normal rabbit IgG. After washing, crosslinks were reversed, and DNA fragments were purified using spin columns. Precipitated DNA was analyzed by PCR with promoter-specific primers (Table 2). Data were normalized to input DNA and presented as fold enrichment relative to IgG control.

### 2.12 RNA fluorescence *in situ* hybridization (FISH)

RNA FISH experiments were conducted in the KGN cells using a specific SNHG12 FISH probe synthesized by RiboBio (Guangzhou, China). Cells were fixed with 4% paraformaldehyde, permeabilized with 0.1% Triton X-100, and subsequently incubated with hybridization buffer (50% formamide, 10% dextran sulfate, and 2×SSC) containing the SNHG12 probe overnight at 37°C. Post-hybridization washes were performed with 50% formamide/2 × SSC for 10 min at 42°C, followed by 1 × SSC for 10 min at 42°C. Nuclei were counterstained with DAPI (Beyotime Biotechnology), and the slides were observed under a microscope (Olympus, Japan).

### 2.13 Flow cytometry

Cells from each experimental group were collected, washed twice with PBS, and stained using an Annexin V-FITC/PI Apoptosis Detection Kit (ABclonal, China) at room temperature for 15 min in the dark. Flow cytometry analysis was performed using a Beckman CytoFLEX flow cytometer. The data were analyzed with FlowJo software (Tree Star, Inc., United States).

### 2.14 Oral glucose tolerance test (OGTT)

For OGTT assay, mice were fasted for 8 h with free access to water. Blood glucose levels were measured using a blood glucose meter (ONETOUCH Ultra Vue, China) via tail vein sampling. Glucose levels were measured after fasting, followed by oral administration of glucose (2 g/kg body weight). Subsequent blood glucose measurements were taken at 15, 30, 60, 90, and 120 min post-administration. The total area under the curve (AUC) for glucose response was calculated using GraphPad Prism 6.0 software.

### 2.15 Glucose uptake and lactate level measurements

Cells and culture medium were collected, and glucose uptake ability and lactic acid levels were evaluated using commercial assay kits (Nanjing Jiancheng Bioengineering Institute, China). For glucose uptake analysis, the culture medium was collected and glucose concentration was determined using a glucose oxidase method assay kit (Cat# A154-1-1). For lactate measurement, the conditioned medium was analyzed using a lactate dehydrogenase (LDH)-based assay kit (Cat# A020-2-2). Absorbance was measured at 505 nm (glucose) and 440 nm (lactate) using a microplate reader.

### 2.16 Statistical analysis

Statistical analyses were performed using SPSS 22.0 statistical software (IBM SPSS, United States). Differences between two groups and multiple groups were compared using the two-tailed Student’s *t*-test and one-way ANOVA respectively. Correlation analysis was evaluated using Spearman’s correlation coefficient. *P* < 0.05 was considered statistically significant.

## 3 Results

### 3.1 SNHG12 is downregulated in GCs of PCOS patients and associated with glycolytic dysfunction

**FIGURE 1:**
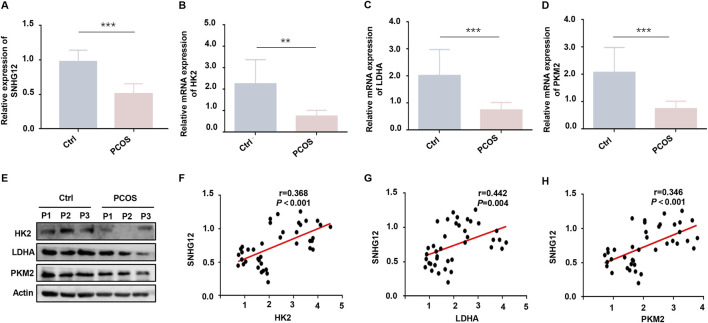
SNHG12 is downregulated in GCs of PCOS patients and associates with glycolytic dysfunction. **(A)** Relative mRNA expression of SNHG12 in GCs from PCOS patients (*n* = 20) and healthy controls (*n* = 20). **(B–D)** RT-PCR analysis of HK2, LDHA and PKM2 mRNA levels in GCs from PCOS patients and controls. **(E)** Western blot analysis of HK2, LDHA and PKM2 protein levels in GCs from PCOS patients and controls. **(F–H)** Correlation analysis between the SNHG12 expression and key glycolytic enzymes (HK2, LDHA and PKM2) in GCs. ^*^ *P* < 0.05, ^**^ *P* < 0.01, and ^***^ *P* < 0.001.

To explore the potential role of SNHG12 in the pathogenesis of PCOS, we first analyzed its expression in obtained GCs samples using RT-PCR. Our results revealed significant downregulation of SNHG12 in PCOS patients when compared to healthy controls (Figure 1A). Given the established importance of glycolytic activity in GCs for oocyte development (Richani et al., 2021) and the reported glycolytic impairment in the GCs of PCOS (Zhang et al., 2022), we subsequently examined the expression of key glycolytic regulators, including HK2, LDHA and PKM2 (Patsoukis et al., 2015). RT-PCR and Western blot analyses confirmed significantly reduced expression of HK2, LDHA and PKM2 levels in the PCOS group when compared with controls (Figures 1B–E).

Emerging evidence indicates that SNHGs modulate glycolytic pathways across multiple cell types (Huang et al., 2022; Li et al., 2024). To further assess the relationship between SNHG12 and glycolysis-related genes, correlation analysis was utilized and the results showed that SNHG12 expression was positively correlated with the levels of HK2 (*r* = 0.368, *P* < 0.001; Figure 1F), LDHA (*r* = 0.442, *P* = 0.004; Figure 1G), and PKM2 (*r* = 0.346, *P* < 0.001; Figure 1H), suggesting that SNHG12 may be closely linked to glycolytic activity in the GCs of PCOS. Additionally, relevant clinical data indicated that SNHG12 expression was negatively correlated with body mass index (BMI) (*r* = −0.409, *P* = 0.009), serum testosterone levels (*r* = −0.687, *P* < 0.001), anti-Mullerian hormone (AMH) (*r* = −0.454, *P* = 0.021), and antral follicle count (AFC) (*r* = −0.565, *P* = 0.001) (Supplementary Figures S1A–D). These results establish SNHG12 as a potential contributor to PCOS pathogenesis.

### 3.2 SNHG12 exerts a significant effect on glycolysis of KGN cells

**FIGURE 2:**
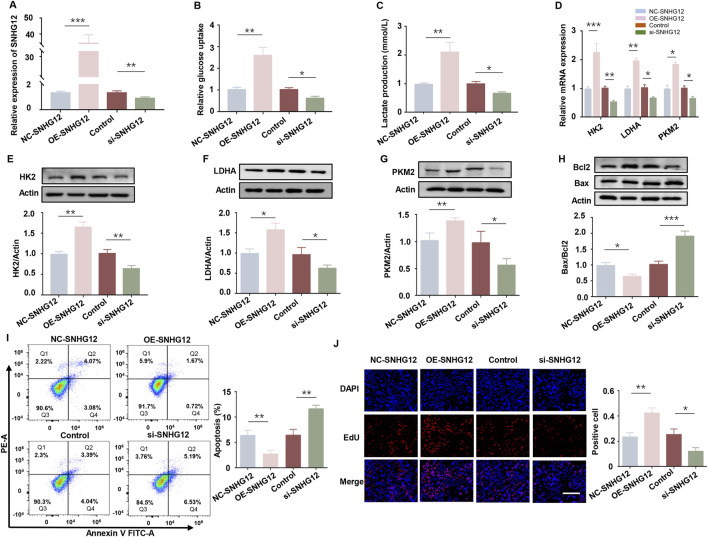
SNHG12 exerts a significant effect on glycolysis of KGN cells. **(A)** mRNA levels of SNHG12 in KGN cells transfected with OE-SNHG12 or si-SNHG12. **(B,C)** Glucose uptake and lactate production in KGN cells transfected with OE-SNHG12 or si-SNHG12. **(D)** Relative mRNA expression of HK2, LDHA and PKM2 in KGN cells treated with SNHG12 overexpression or knockdown. **(E–G)** Protein levels of HK2, LDHA and PKM2 in KGN cells treated with SNHG12 overexpression or knockdown. **(H)** Western blot analysis of the Bax/Bcl2 ratio in KGN cells transfected with OE-SNHG12 or si-SNHG12. **(I)** Apoptosis of KGN cells in each group was detected by flow cytometry. **(J)** Representative images of EdU assay results in different groups. Scale bar = 50 μm. *n* = 3, ^*^ *P* < 0.05, ^**^ *P* < 0.01, and ^***^ *P* < 0.001.

To investigate the effects of SNHG12 on glycolysis *in vitro*, KGN cells were transfected with either an SNHG12 overexpression vector (OE-SNHG12) or small interfering RNAs targeting SNHG12 (si-SNHG12). RT-PCR analysis verified the efficiency (Figure 2A). Glucose uptake and lactate production were significantly elevated in the OE-SNHG12 group but suppressed by SNHG12 knockdown compared to the control group (Figures 2B,C). Furthermore, OE-SNHG12 upregulated the mRNA and protein levels of key glycolytic enzymes (HK2, LDHA and PKM2), whereas si-SNHG12 exerted the opposite effects (Figures 2D–G). Given that glycolytic dysfunction is often associated with aberrant proliferation and apoptosis of GCs in PCOS (Cao et al., 2022), we further assessed the function of SNHG12 in regulating the viability of KGN cells. Remarkably, SNHG12 overexpression decreased the Bax/Bcl2 ratio, reduced apoptosis, and increased proliferation. Conversely, SNHG12 knockdown reversed these effects, leading to increased apoptosis and impaired proliferation (Figures 2H–J). These data suggest that SNHG12 plays a critical role in regulating glycolysis and modulating the proliferation and apoptosis of KGN cells.

### 3.3 SNHG12 alleviates metabolic disorders and improves ovarian follicle development in PCOS mice

**FIGURE 3:**
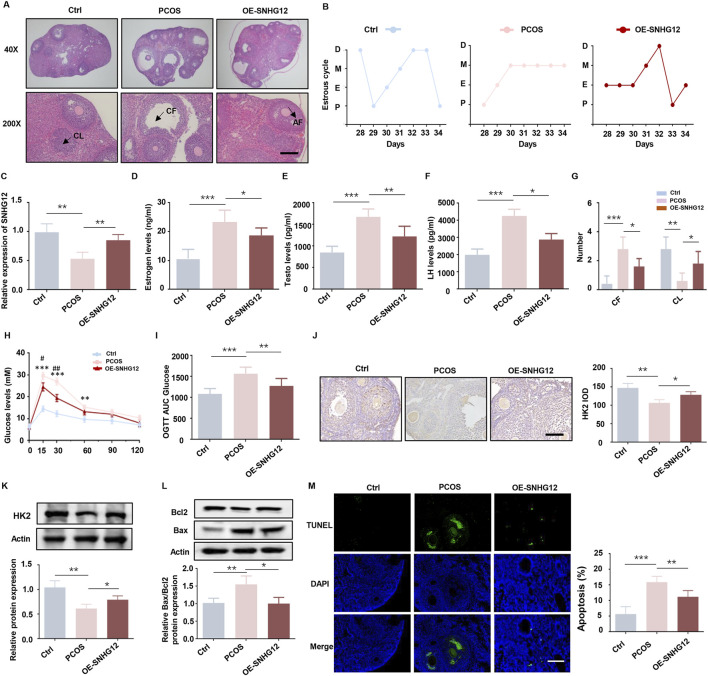
SNHG12 alleviates metabolic disorders and improves ovarian follicle development in PCOS mice. **(A)** Representative HE staining of ovarian sections from control (*n* = 6), PCOS (*n* = 6) and OE-SNHG12 groups (magnification: 40× and 200×). Scale bar: 50 µm. **(B)** Cytological analysis of vaginal smears in control, PCOS and OE-SNHG12 groups. **(C)** Relative mRNA expression of SNHG12 in ovarian tissues in the indicated groups. **(D–F)** Serum levels of estradiol (E_2_), testosterone (T), and luteinizing hormone (LH) in the indicated groups. **(G)** The number of cystic follicles (CF) in the ovaries of the mice in the indicated groups. **(H,I)** Glucose tolerance tests (GTT) and area under the curve (AUC) analysis in the experimental mice. **(J)** IHC staining of HK2 in ovarian tissues from the indicated groups (magnification: 400×). Scale bar: 50 µm. **(K)** Western blot analysis of HK2 expression in ovarian tissues from each group. **(L)** Western blot analysis of the Bax/Bcl2 ratio in ovarian tissues from each group. **(M)** Representative images of TUNEL assay results in mouse ovaries from the indicated groups. Scale bar: 50 µm ^*^ *P* < 0.05, ^**^ *P* < 0.01, and ^***^ *P* < 0.001.

Building upon our *in vitro* findings, we established a DHEA-induced PCOS mouse model to explore SNHG12’s role *in vivo*. The successful establishment of the PCOS model was confirmed by the presence of increased cystic follicles and disrupted estrus cycles (Figures 3A,B). RT-PCR analysis indicated that SNHG12 expression in ovarian tissues was significantly elevated in the OE-SNHG12 group compared to the PCOS group, indicating effective SNHG12 overexpression *in vivo* (Figure 3C). As indicated in Figures 3D–F, serum levels of sex hormones, including estradiol (E_2_), testosterone (T) and luteinizing hormone (LH) were distinctly increased in the PCOS group but significantly decreased following OE-SNHG12 adenovirus administration. Ovarian histology revealed that OE-SNHG12 treatment reduced cystic follicles (CF) and increased corpora lutea (CL) compared to the PCOS group (Figure 3G). In glucose tolerance tests (GTT), PCOS mice displayed impaired glucose metabolism, but OE-SNHG12 significantly improved this phenotype (Figures 3H,I). Furthermore, immunohistochemical (IHC) analysis indicated weaker HK2 protein expression in the PCOS group, which was restored by SNHG12 overexpression (Figures 3J,K). Western blot analysis showed that SNHG12 overexpression significantly decreased the pro-apoptotic Bax/Bcl2 ratio in ovarian GCs (Figure 3L), a finding corroborated by reduced TUNEL-positive cells in the OE-SNHG12 group (Figure 3M). Overall, these findings suggest that SNHG12 alleviates metabolic disorders and promotes ovarian follicle development in PCOS mice.

### 3.4 PTEN is a key downstream target of SNHG12

**FIGURE 4:**
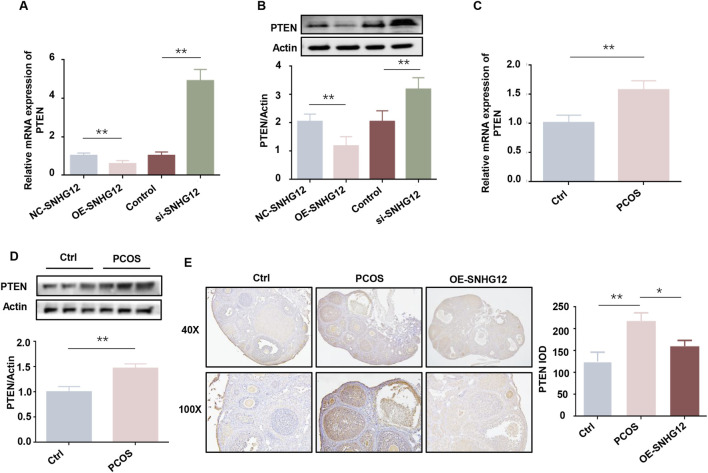
PTEN is a key downstream target of SNHG12. **(A)** mRNA levels of PTEN in KGN cells transfected with OE-SNHG12 or si-SNHG12. **(B)** Protein levels of PTEN in KGN cells transfected with OE-SNHG12 or si-SNHG12. **(C,D)** mRNA and protein levels of PTEN in GCs from PCOS patients (*n* = 20) and healthy controls (*n* = 20). **(E)** IHC staining of PTEN in ovarian tissues from control and PCOS mice (magnification: 40× and 100×). Scale bar: 50 µm ^*^ *P* < 0.05, ^**^ *P* < 0.01, and ^***^ *P* < 0.001.

PTEN functions as a metabolic critical regulator, modulating glycolysis and other metabolic processes that are indispensable for cell growth (Ortega-Molina and Serrano, 2013). Previous studies have highlighted the pivotal role of PTEN in regulating GCs proliferation and apoptosis in PCOS (Gao et al., 2021; Liu et al., 2020). Given PTEN’s role in metabolism and GCs function, we hypothesized that PTEN might mediate SNHG12’s regulation of glycolysis. As shown in Figure 4A, SNHG12 overexpression significantly decreased PTEN mRNA levels in KGN cells, while SNHG12 knockdown increased PTEN mRNA expression. Western blot analysis confirmed corresponding changes at the protein level (Figure 4B). Notably, PTEN mRNA and protein levels were elevated in GCs from PCOS patients compared with controls (Figures 4C,D). IHC analysis of ovarian tissues from DHEA-induced PCOS mice also revealed a similar trend, with decreased PTEN expression following OE-SNHG12 adenovirus treatment (Figure 4E). These results collectively established PTEN as a key downstream target of SNHG12.

### 3.5 SNHG12 regulates glycolysis in KGN cells partially through PTEN

**FIGURE 5:**
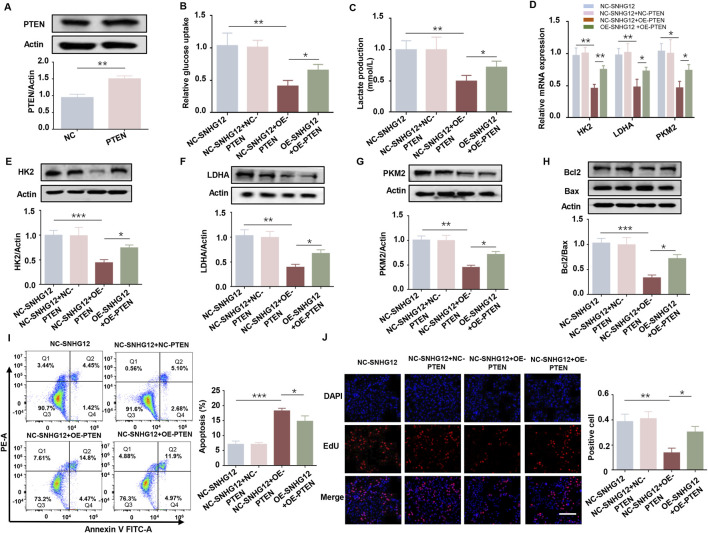
SNHG12 regulates glycolysis in KGN cells partially through PTEN. **(A)** Western blot analysis confirming efficiency of PTEN overexpression in KGN cells. **(B,C)** Glucose uptake and lactate production in KGN cells transfected with OE-SNHG12 or PTEN. **(D)** Relative mRNA expression of HK2, LDHA and PKM2 in the indicated KGN cells. **(E–G)** Protein levels of HK2, LDHA and PKM2 in the indicated KGN cells. **(H)** Western blot analysis of the Bax/Bcl2 ratio in the indicated KGN cells. **(I)** Apoptosis of KGN cells in each group was detected by flow cytometry. **(J)** Representative images of EdU assay results in the indicated KGN cells. Scale bar = 50 μm. n = 3, ^*^ *P* < 0.05, ^**^ *P* < 0.01, and ^***^ *P* < 0.001.

To further investigate whether PTEN mediates the functional effects of SNHG12, we overexpressed PTEN in KGN cells. The efficiency of PTEN overexpression was confirmed by Western blot (Figure 5A). Glucose consumption and lactate production were significantly reduced in the OE-PTEN group, while overexpression of SNHG12 reversed these effects (Figures 5B,C). Consistently, RT-PCR and Western blot analysis of key glycolysis-related genes (HK2, LDHA and PKM2) showed similar results, further supporting the role of SNHG12 in regulating glycolysis through PTEN (Figures 5D–G). Furthermore, PTEN overexpression promoted apoptosis and inhibited the proliferation in KGN cells, while SNHG12 overexpression partly counteracted the PTEN-induced effects on proliferation and apoptosis (Figures 5H–J). These findings indicate that SNHG12 regulates glycolysis in KGN cells, at least in part, by modulating PTEN expression.

### 3.6 SNHG12 promotes glycolysis in KGN cells by interacting with HMGB1

To test whether SNHG12 directly binds to PTEN or modulates its promoter activity, we performed RNA pulldown and luciferase reporter assays. The RNA pulldown assay revealed no significant difference in PTEN expression compared to the antisense group (Supplementary Figure S2A). Similarly, the luciferase reporter assay confirmed that SNHG12 had no measurable effects on PTEN promoter activity (Supplementary Figure S2B). These results indicate that SNHG12 regulates PTEN expression through mechanisms independent of direct promoter interactions.

**FIGURE 6:**
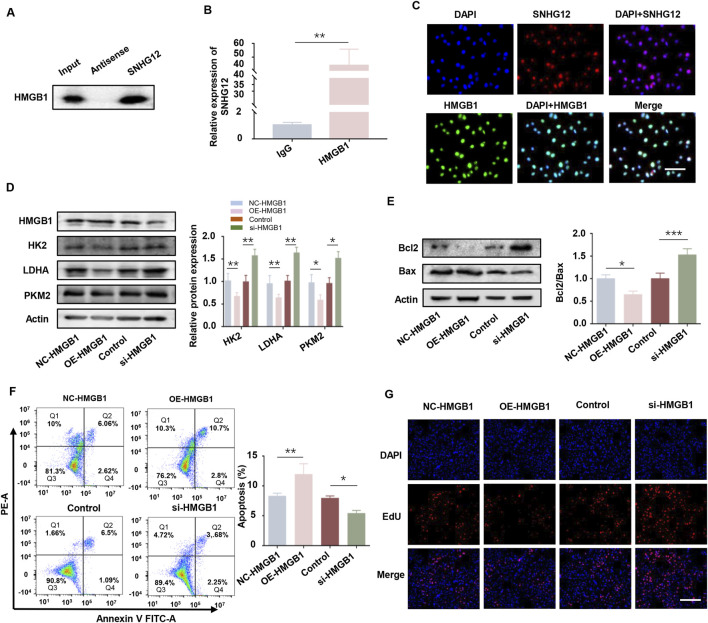
SNHG12 promotes glycolysis in KGN cells by interacting with HMGB1. **(A,B)** RNA pull-down experiment and RIP assays validating the interaction between SNHG12 and HMGB1. **(C)** Immunofluorescence analysis showing co-location of SNHG12 with HMGB1 in the nucleus of KGN cells. Scale bar: 20 µm. **(D)** Protein levels of HK2, LDHA and PKM2 in KGN cells with HMGB1 overexpression or knockdown. **(E)** Western blot analysis of the Bax/Bcl2 ratio in the aforementioned KGN cells. **(F)** Flow cytometry analysis of apoptosis in the aforementioned KGN cells. **(G)** Representative images of EdU assay results in the aforementioned KGN cells. Scale bar = 50 μm. *n* = 3, ^*^ *P* < 0.05, ^**^ *P* < 0.01, and ^***^ *P* < 0.001.

Mounting evidence demonstrates that lncRNAs often mediate their biological functions by interacting with proteins (Luo et al., 2021; Wang et al., 2021). HMGB1 is a non-histone chromosomal protein implicated in PCOS-related glucose metabolism defects and has been reported to interact with lncRNAs (Li et al., 2023; Lou et al., 2021). Based on this, we hypothesized that SNHG12 might affect glycolysis in GCs via interaction with HMGB1. RPISeq bioinformatics analysis predicted a high-probability interaction between SNHG12 and HMGB1 (SVM score = 0.96, RF score = 0.8; Supplementary Figure S2C). Subsequent RIP and RNA pulldown assays validated this interaction in KGN cells (Figures 6A,B). Immunofluorescence staining revealed co-localization of SNHG12 and HMGB1 in the nucleus of KGN cells (Figure 6C), supporting their potential role in transcriptional regulation. Functionally, upregulation of HMGB1 significantly suppressed the expression of glycolytic genes (HK2, LDHA, and PKM2), while HMGB1 knockdown showed the opposite trend (Figure 6D). Western blotting, flow cytometry and EdU assays revealed that HMGB1 overexpression enhanced apoptosis and inhibited proliferation in KGN cells, whereas HMGB1 knockdown yielded the reverse results (Figures 6E–G). Taken together, these results suggest that SNHG12 enhances glycolysis in KGN cells by binding to HMGB1.

### 3.7 SNHG12 suppresses PTEN transcription by competitively binding to HMGB1

**FIGURE 7:**
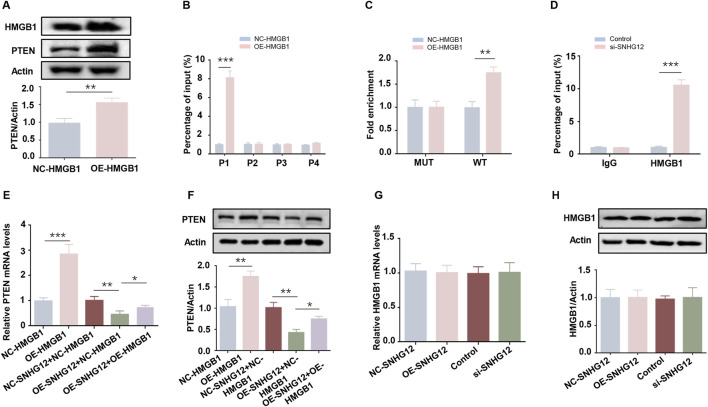
SNHG12 suppresses PTEN transcription by competitively binding to HMGB1. **(A)** Western blot analysis of the PTEN in HMGB1-overexpressing KGN cells. **(B)** ChIP-PCR assay of binding of HMGB1 to PTEN promoter region in KGN cells. **(C)** Luciferase reporter assays of KGN cells overexpressing HMGB1 and transfected with reporter plasmids containing WT and MUT PTEN promoter. **(D)** ChIP assays performed in KGN cells using HMGB1 antibodies or IgG. **(E,F)** RT-PCR and Western blot analyses of PTEN expression levels after HMGB1 upregulation in SNHG12-knockdown KGN cells. **(G,H)** mRNA and protein levels of HMGB1 in KGN cells were determined by RT-PCR and Western blot when treated with OE-SNHG12 or si-SNHG12. *n* = 3, ^*^ *P* < 0.05, ^**^ *P* < 0.01, ^***^ *P* < 0.001.

As a well-known negative regulator of glycolysis, PTEN has been reported to be regulated by HMGB1 (Zhou et al., 2019). To elucidate the relationship between HMGB1 and PTEN in KGN cells, we overexpressed HMGB1 and observed a significant upregulation of PTEN (Figure 7A), whereas PTEN knockdown did not alter HMGB1 expression (Supplementary Figure S3). To determine whether HMGB1 directly modulates PTEN transcription, the JASPAR database was used to predict four putative HMGB1 binding sites in the PTEN promoter (Supplementary Figure S4). ChIP-PCR analysis in KGN cells identified the P1 site as the primary HMGB1 binding region (Figure 7B). The luciferase reporter assay demonstrated that HMGB1 overexpression increased the activity of the wild-type (WT) PTEN promoter but failed to activate the P1-mutated (MUT) promoter (Figure 7C). These results suggest that HMGB1 directly modulates PTEN transcription in KGN cells.

Next, we examined whether SNHG12 affects the binding of HMGB1 to the PTEN promoter. ChIP assays revealed that SNHG12 depletion promoted HMGB1 binding to the P1 site of the PTEN promoter (Figure 7D), suggesting that SNHG12 competitively sequesters HMGB1. Rescue experiments further verified that the SNHG12-mediated reduction in both PTEN mRNA and protein levels was reversed by HMGB1 overexpression (Figures 7E,F). Notably, HMGB1 mRNA and protein levels remained unchanged in both the SNHG12 knockdown and overexpression groups (Figures 7G,H), thereby excluding the possibility that SNHG12 inhibited PTEN transcription through downregulation of HMGB1 expression. These results demonstrate that SNHG12 suppresses the transcription of PTEN by competitively binding to HMGB1.

## 4 Discussion

**FIGURE 8:**
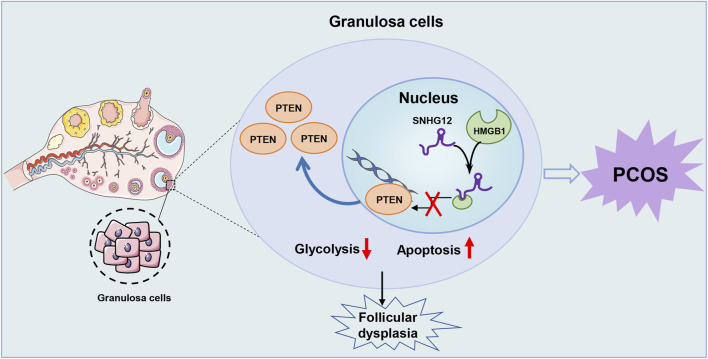
Schematic illustration of the role of SNHG12 in GCs during PCOS. Our study demonstrates that SNHG12 regulates glycolysis in GCs by disrupting the interaction between HMGB1 and the PTEN promoter, thereby modulating PTEN transcription and contributing to the pathophysiology of PCOS.

Cellular glycolysis is a fundamental biological process essential for energy production, apoptosis, and proliferation, including GCs (Pokharel et al., 2023; Vaupel and Multhoff, 2021). As a key component of glucose metabolism, glycolysis serves as the predominant metabolic pattern in GCs, enabling the rapid production of pyruvate and lactate to support anabolic processes (Richani et al., 2021). In this study, we identified SNHG12, a lncRNA downregulated in GCs of PCOS patients, as a molecule that competitively disrupts the interaction between HMGB1 and the PTEN promoter, thereby regulating the glycolytic pathway in GCs. This regulatory mechanism contributes to apoptosis and follicular dysplasia (Figure 8), highlighting the critical role of SNHG12 in PCOS-associated follicular dysfunction through glycolytic regulation.

LncRNAs have emerged as critical regulators in glycolysis during physiological processes by directly or indirectly activating dominant glycolytic enzymes (Duan et al., 2024; Xu et al., 2022; Zhu et al., 2022). Consistent with this paradigm, our study demonstrates that SNHG12 participates in glycolysis-dependent regulation. Specifically, we observed that SNHG12 expression was significantly downregulated in PCOS GCs and positively correlated with glycolytic markers (HK2, LDHA, PKM2). Functional experiments demonstrated that SNHG12 silencing suppressed glycolysis, decreased lactate production, and disrupted the follicular development. It is well established that aberrant apoptosis of GCs is a major contributor to follicular atresia (Zhang et al., 2021). In our study, we observed that SNHG12 deficiency exacerbated GCs apoptosis through glycolytic suppression, ultimately contributing to follicular dysplasia.

PTEN (phosphatase and tensin homologue) is a tumor suppressor gene that plays a crucial role in regulating cell growth and signal transduction (Huang et al., 2017). Elevated PTEN expression has been observed in PCOS patients and is implicated in the regulation of ovarian follicle growth and oocyte maturation (Liu et al., 2020; Namli Kalem et al., 2023). In addition, studies have shown that lncRNAs can modulate PTEN expression directly or indirectly (Li et al., 2018). Consistent with these findings, our study revealed that PTEN expression was significantly upregulated in PCOS samples compared to those in healthy controls. Functional experiments demonstrated that SNHG12 overexpression downregulated PTEN mRNA and protein levels, while SNHG12 knockdown produced the opposite trends. Furthermore, the role of PTEN as a metabolic regulator has gained much attention, with evidence suggesting its involvement in glycolysis across various cell types (Chatterjee et al., 2019; Endicott and Miller, 2024). To further investigate this, we transfected a PTEN-overexpressing plasmid into KGN cells, which revealed that SNHG12 overexpression could partially rescue PTEN-induced glycolytic suppression and apoptosis. These findings demonstrate that SNHG12 regulates the glycolytic activity of GCs, at least in part, through PTEN. However, the precise molecular mechanisms by which SNHG12 modulates PTEN require future validation.

As a non-histone chromosome structural protein, HMGB1 regulates various nuclear functions, including transcription, DNA repair, and genome stability maintenance (Travers, 2003). HMGB1 dysregulation has been extensively implicated in PCOS pathophysiology (Zhang et al., 2020; Zhu et al., 2020), and recent studies have linked elevated serum HMGB1 to impaired glucose metabolism in PCOS patients (Moghetti et al., 2023). Furthermore, HMGB1 has been identified as a crucial target of lncRNAs in multiple cell types (Kou et al., 2023; Zeng et al., 2023). Building on these findings, our study discovered that SNHG12 directly interacts with HMGB1 and co-localizes with HMGB1 in the nucleus of KGN cells. These results align with previous studies showing that nucleus-retained lncRNAs can influence gene transcription and epigenetic regulation (Lin et al., 2023; Zhuo et al., 2019). Notably, Xuan et al. reported that cytoplasmic SNHG12 regulates proliferation and apoptosis through miRNA sponging in insulin-treated KGN cells (Xuan et al., 2024), a phenomenon that may reflect insulin-induced nuclear-to-cytoplasmic translocation of lncRNAs (Holly et al., 2019). Additionally, we found that HMGB1 overexpression reversed the SNHG12-mediated suppression of PTEN expression, even though HMGB1 levels remained unchanged upon SNHG12 interference. These results uncover an HMGB1-dependent nuclear mechanism by which SNHG12 contributes to PCOS progression, providing valuable insights into the therapeutic avenue for PCOS patients. However, the present study has several limitations. First, *in vitro* experiments were conducted using cell lines rather than primary cells. Second, the metabolic activity of GCs following SNHG12 treatment in PCOS mouse models warrants further exploration. Finally, alternative regulatory mechanisms of SNHG12 on PTEN expression, including potential ceRNA networks and epigenetic modifications, require further investigation.

In conclusion, we have, for the first time, demonstrated that SNHG12 is involved in the regulation of glycolysis in GCs, thereby influencing the follicular dysplasia in PCOS. Mechanistically, SNHG12 competitively binds to HMGB1, disrupting its interaction with the PTEN promoter and subsequently modulating PTEN transcription. These findings provide novel insights into the role and mechanisms of SNHG12 in PCOS, highlighting its potential as a therapeutic target.

## Data Availability

The original contributions presented in the study are included in the article/Supplementary Material, further inquiries can be directed to the corresponding authors.
