# Supplementary material for: SNHG12 downregulation induces follicular dysplasia by modulating the glycolysis of granulosa cell in polycystic ovary syndrome
Source: Front Cell Dev Biol. 2025 May 23;13:1585987. doi: 10.3389/fcell.2025.1585987 (PMC12141224; doi:10.3389/fcell.2025.1585987)
Supplement: Supplementary file 1 [file Supplementaryfile1.pdf]

Supplementary materials

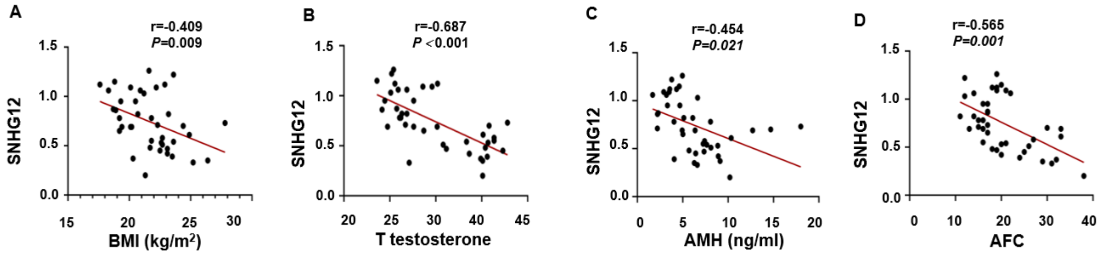

Figure S1. Correlation analysis between SNHG12 and relevant clinical data.

Association of SNHG12 expression with BMI (A), Testosterone (B), AMH (C) and AFC (D) in PCOS patients (n=20) and healthy controls (n=20).

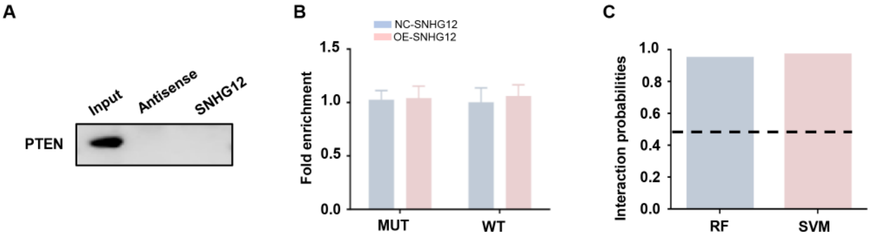

Figure S2. The interaction between SNHG12 and PTEN.

(A) RNA pulldown confirmed the interaction between SNHG12 and PTEN proteins in KGN cells. (B) Luciferase reporter assays of KGN cells overexpressing SNHG12 and transfected with reporter plasmids containing WT and MUT PTEN promoter. (C)The interaction between SNHG12 and HMGB1 predicted by RNA-Protein Interaction Prediction. RF: Random Forest; SVM: Support Vector Machine.

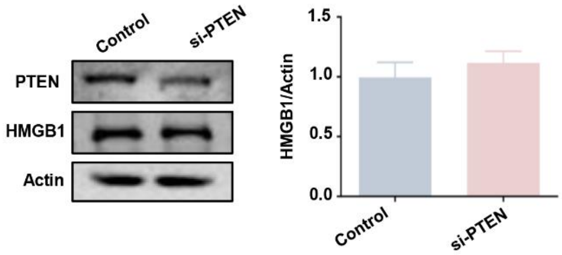

Figure S3. The effect of PTEN on the expression of HMGB1.

Western blot analysis of the HMGB1 in PTEN-overexpression KGN cells.

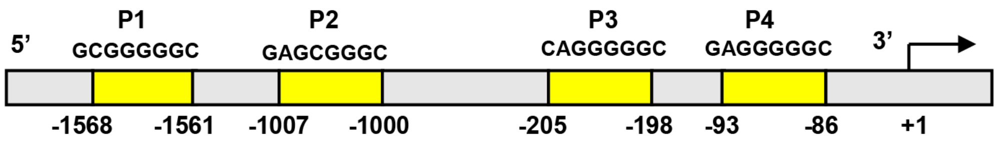

Figure S4. A direct binding of HMGB1 with the PTEN promoter.

Diagram for seed regions of 4 predicted HMGB1-binding sites in PTEN promoter. P1: the motif at nt -1568 to -1561; P2 the motif at nt -1007 to -1000; P3: the motif at nt -205 to -198; P4 the motif at nt -93 to -86 of the PTEN promoter.
